# Young fibroblast-derived exosomal microRNA-125b transfers beneficial effects on aged cutaneous wound healing

**DOI:** 10.1186/s12951-022-01348-2

**Published:** 2022-03-19

**Authors:** Wenzheng Xia, Minxiong Li, Xingyu Jiang, Xin Huang, Shuchen Gu, Jiaqi Ye, Liaoxiang Zhu, Meng Hou, Tao Zan

**Affiliations:** 1 Department of Plastic and Reconstructive Surgery, Shanghai Ninth People’s Hospital, Shanghai Jiao Tong University School of Medicine, 200011 Shanghai, China; 2 School of Chinese Materia Medica, Nanjing University of Chinese Medicine, Nanjing, China; 3 Department of Radiation Oncology, First Affiliated Hospital, Wenzhou Medical University, No. 2 Fuxue Lane, 325000 Wenzhou, People’s Republic of China

**Keywords:** Wound healing, Exosome induced microRNA delivery, Senescence, Fibroblast to myofibroblast transition, ECM deposition

## Abstract

Aged skin wounds heal poorly, resulting in medical, economic, and social burdens posed by nonhealing wounds. Age-related defects in repair are associated with reduced myofibroblasts and dysfunctional extracellular matrix (ECM) deposition. Bidirectional cell-cell communication involving exosome-borne cargo such as micro RNAs (miRs) has emerged as a critical mechanism for wound healing and aged tissue regeneration. Here we report that at the wound edge, aged fibroblasts display reduced migration and differentiation into myofibroblasts, with impaired ECM deposition, when compared with young tissue. Proper activation of fibroblasts to myofibroblasts may alleviate age-related defects in wound healing. Herein, an exosome-guided cell technique was performed to induce effective wound healing. Supplementing wounds with exosomes isolated from young mouse wound-edge fibroblasts (exosomes^Young^) significantly improved the abundance of myofibroblasts and wound healing in aged mice and caused fibroblasts to migrate and transition to myofibroblasts in vitro. To determine the underlying mechanism, we found that exosomal transfer of miR-125b to fibroblasts inhibited sirtuin 7 (Sirt7), thus accelerating myofibroblast differentiation and wound healing in aged mice. Notably, after epidermal inhibition of miR-125b or overexpression of Sirt7 in fibroblasts, migration and myofibroblast transition were perturbed. Our findings thus reveal that miR-125b is transferred through exosomes from young fibroblasts to old fibroblasts contributes to promoting fibroblast migration and transition to counteract aging, suggesting a potential avenue for anti-aging interventions in wound healing.

**Graphical Abstract:**

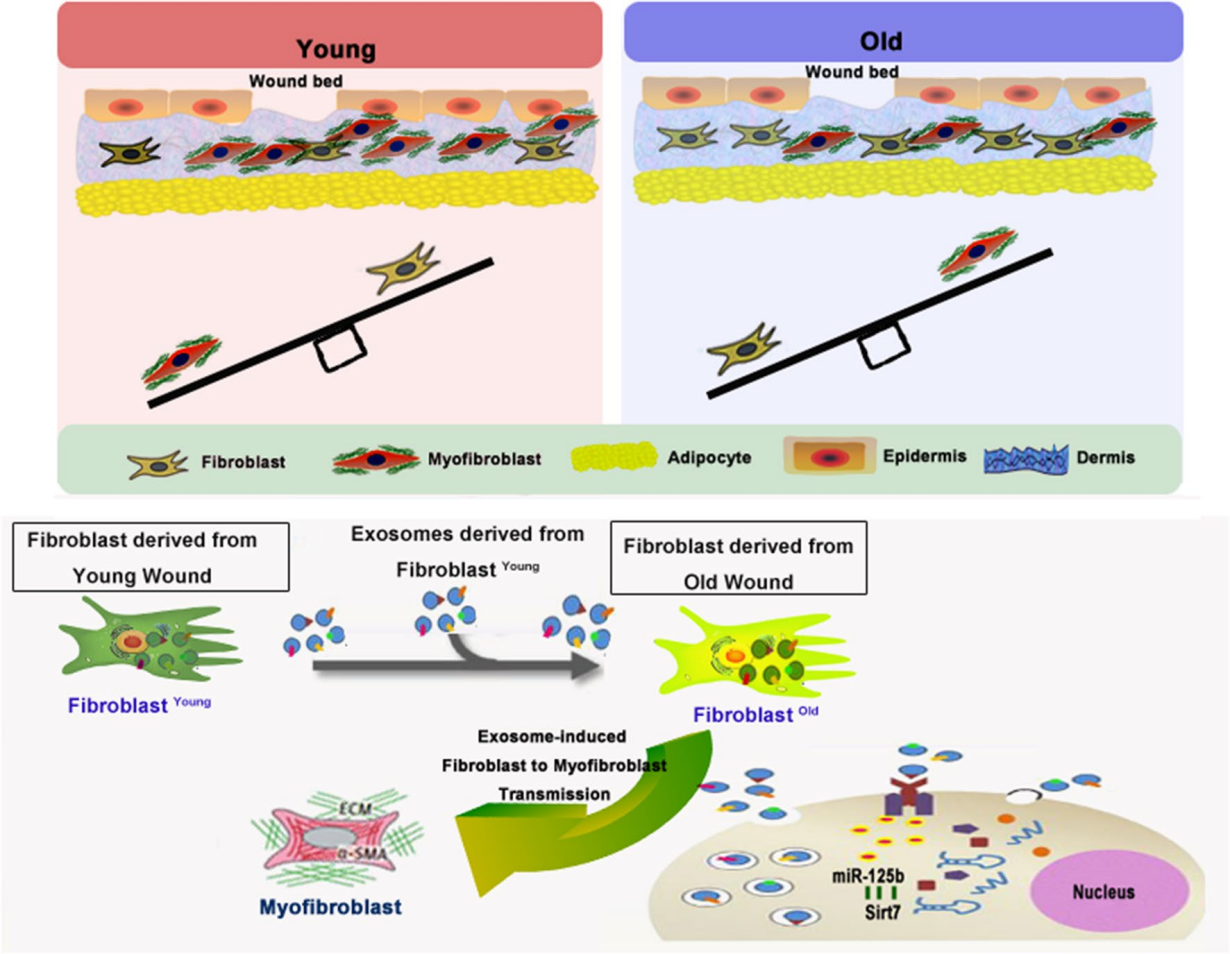

**Supplementary Information:**

The online version contains supplementary material available at 10.1186/s12951-022-01348-2.

## Introduction

The repair of wounds is one of the most complex biological processes that occurs throughout life [1]. This process requires a long healing cycle during which severe structural and functional damage or further infection sometimes occur [2]. The list of risk factors for defects in wound healing is still evolving, but indicates an intricate relationship between local and systemic factors such as age, diabetes, and systemic inflammation [3, 4]. Age is one of the major risk factors for impaired wound healing [5]. Age-related defects in wound healing contribute to a variety of health complications, even to decreased lifespan. Despite its importance, however, the molecular mechanisms underlying age-related defects in wound repair are not well understood, impeding the prospects for therapeutic advances.

There are three classic stages of wound repair: inflammation, new tissue formation and remodeling [1]. A crucial stage during the wound-healing process is new tissue formation, which occurs 2–10 days after injury. During this stage, most cells from the previous stage of repair migrate from the wound and ECM is deposited, myofibroblasts produce ECM molecules for tissue resilience and strength, and altered ECM deposition can lead to tissue dysfunction and disease [6–8]. In general, during the wound-repair process, fibroblasts, which are attracted from the edge of the wound, differentiate into myofibroblasts and ultimately form the ECM to induce the healing process [9]. Organ and tissue senescence have been associated with cellular defects in migration, proliferation, and differentiation in the regeneration process [10]. Age-related defects in repair are associated with reduced myofibroblasts and dysfunctional ECM deposition [11]. The exploration of mechanisms underlying fibroblast migration and their transition to myofibroblasts in the aged wound bed may help find new therapeutic targets to repair aged-related defects in wound healing.

Transfer of blood from young animals by administration of young blood plasma, which has been shown to improve regenerative capacity in aged mice [12, 13], motivated us to explore whether such a transfer would make sense in the aged-healing process. Exosomes, nano-sized membrane-enclosed vesicles released by cells into extracellular spaces in living organisms or cell culture medium, play an important role in cellular communication [14]. Proteins, lipids, and genetic material can be delivered to induce cellular communication [15]. Exosomes are of therapeutic interest because they are deregulated in diseases and they could be harnessed to deliver drugs to target cells [16, 17]. Exosomal transfer of therapeutic effects in wound healing has been discovered to optimize fibroblast functions [18]. Given the parallels between the effects of exosomes and youthful regenerative mechanisms, we tested whether exosomes derived from fibroblasts isolated from young wound beds could confer beneficial effects on wound healing in the aged mice.

MicroRNAs (miRs), 20–24 nucleotides in length, which act in post-transcriptional regulation of gene expression, are attracting growing interest in the field of wound healing due to their therapeutic effects [19, 20]. MiRs, one of important antisense oligonucleotides (ASOs), are promising disease altering modalities because they target disease causing genes in a sequence specific manner [21]. Exosome carriers offer unprecedented opportunities for cell-specific controlled delivery of miRs for therapeutic goals [22]. Transfer of miRs via exosomes leading to altered protein expression and phenotypes of recipient cells [23]. For example, exosomes delivering miRs accelerated cutaneous wound healing by promoting fibroblast migration [24]. Local fibroblasts recruited into a wound can then differentiate into myofibroblasts [25], but an insufficient population of myofibroblasts resulted in age-related defective wound healing [8]. MiR-125b, as one of the more important TGF-β profibrotic signaling-targeted miRs, has been shown to be necessary and sufficient for the induction of the fibroblast-to-myofibroblast transition (FMT) [26]. Transfection of miR-125b mimics into fibroblasts resulted in a significantly increased number of α-smooth muscle actin (SMA)-positive myofibroblasts, which are important in ECM deposition [27]. Our previous work revealed that miR-125b was enriched in exosomes derived from fibroblasts isolated from young mice wound beds. However, the functional role of this miR in the induction of FMT-induced exosome-related wound healing has not yet been characterized.

In this study, we investigated he mechanistic role of exosome-delivered miR-125b in aged fibroblast FMT and migration. We then tested the therapeutic potential of miR-125b in age-related defective wound healing. We conclude that young fibroblasts stimulated exosome-induced upregulation of miR-125b in old fibroblasts which could promote both migration and FMT, eventually resulting in accelerated wound healing in older mice.

## Results

### Aging is associated with defects in wound healing and decreasing FMT

**Fig. 1:**
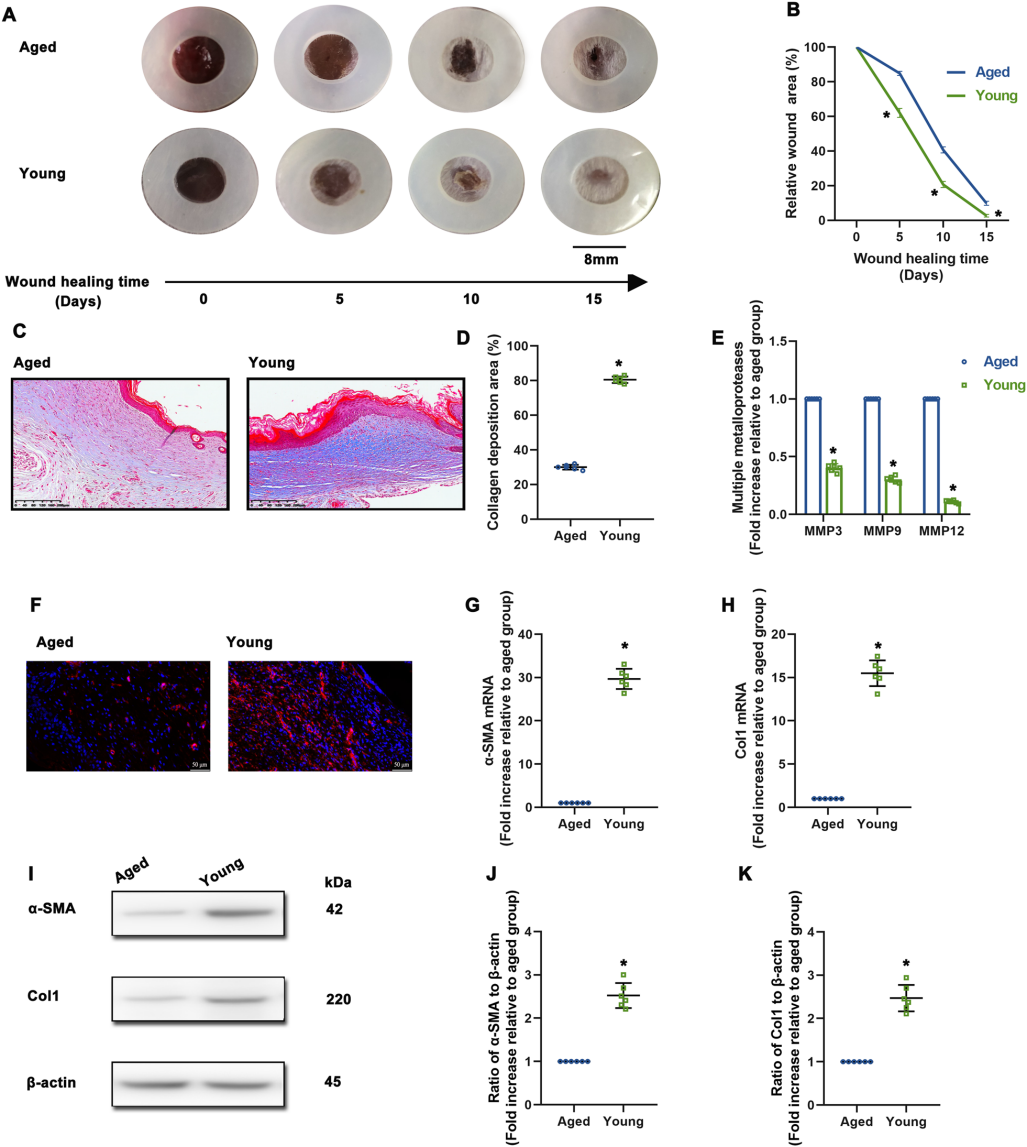
Aging is associated with defects in wound healing and decreasing FMT. **A** Representative images of wound closure in aged and young groups. **B** Relative wound area was calculated. **C** Masson’s trichome staining of wound sections in aged and young groups (scale bar: 200 μm). **D** % of collagen deposition was calculated. **E** MMP3, MMP9 and MMP12 mRNA levels were analyzed using qRT-PCR. **F** Immunofluorescence staining for α-SMA was used to examine the density of myofibroblasts (scale bar: 50 μm). **G**, **H** α-SMA and Col1 mRNA levels were analyzed. **I** Representative results of western blot analysis for α-SMA and Col1. **J**, **K** Analysis of western blot results for α-SMA and Col1. ^*^P < 0.05 versus Aged by Student’s *t*-tests (n = 6)

Aging has negative effects on the regenerative capacity of damaged tissues [28]. First, *in vivo* wound-repair ability of aged and young mice was examined by visual assessment of excisional wound closure. When compared with the young group, the aged group showed defects in wound closure (Fig. 1A, B).

Age-related defects in repair are associated with reduced myofibroblasts and dysfunctional ECM deposition. To gain insights into the etiology of defects in healing, we first identified changes in collagen deposition. Masson’s trichrome staining showed that the aged group had decreased collagen in the wound area compared to young group (Fig. 1C, D). Interestingly, the expression of multiple matrix metalloproteases (MMPs) including MMP3, MMP9 and MMP 12, which are involved in the break down the ECM, was increased in aged mice (Fig. 1E). With respect to FMT, immunofluorescence staining results for α-SMA, which is the most common marker used to identify myofibroblasts, showed that more FMT presented in the wound bed of young mice (Fig. 1F). Meanwhile, within the wound beds of the young mice, the expression of α-SMA and collagen I (Col1) were upregulated compared to the aged group (Fig. 1G–K).

### Exosome ^Young^ -guided phenotypic switch to myofibroblasts accelerated wound healing

**Fig. 2:**
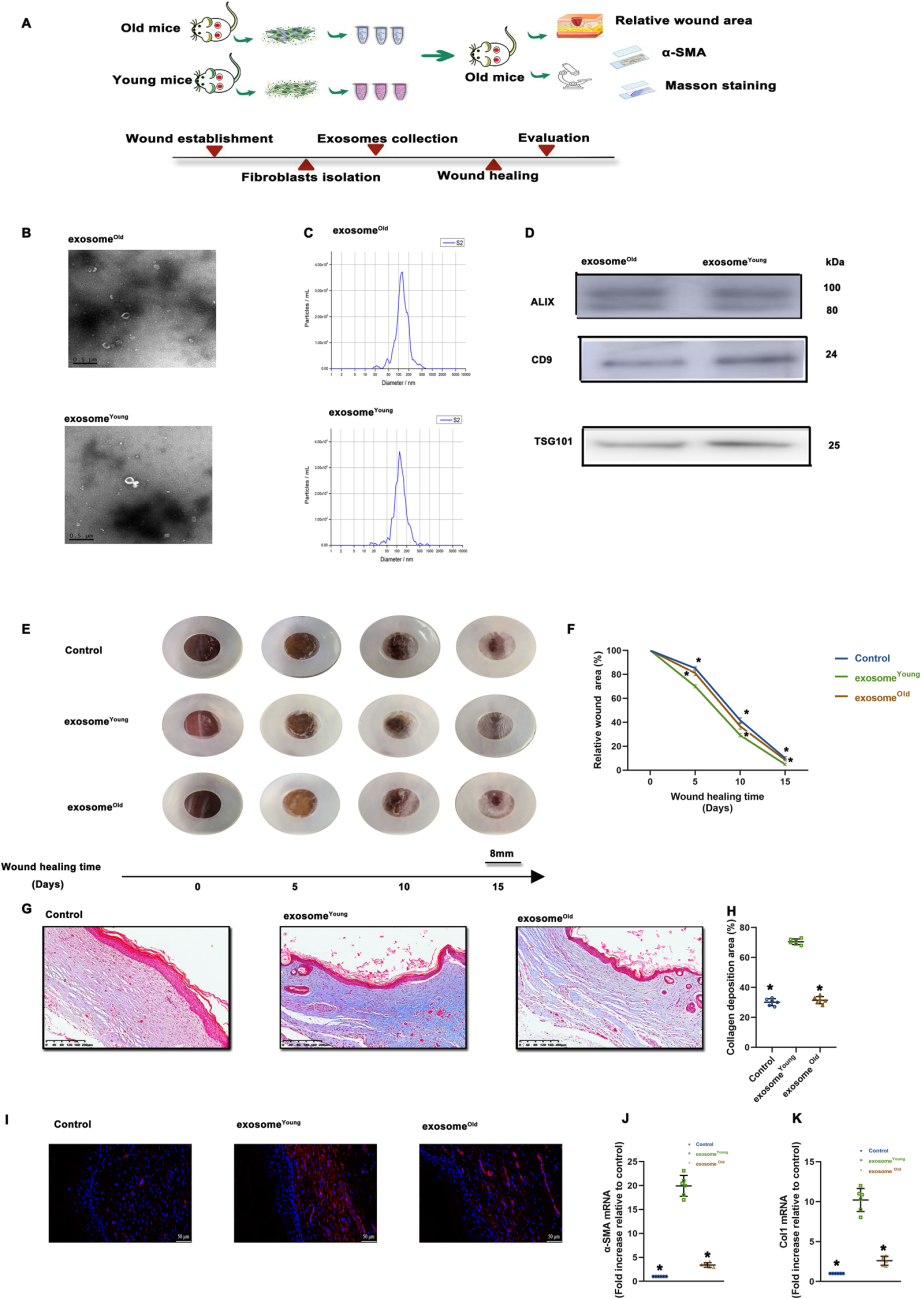
Exosome^Young^ -guided phenotypic switch to myofibroblasts accelerated wound healing. **A** Schematic diagram of mouse treatment. Exosomal collection was confirmed using TEM, NTA and western blot analysis. **B** Representative TEM image. **C** Size range of exosomes validated using NTA. **D** Representative western blot images showing that the exosomal markers ALIX, CD9 and TSG101 were expressed in exosome^Old^ and exosome^Young^. **E** Representative images of wound closure after local injection of saline (Control), exosome^Old^ and exosome^Young^. **F** Relative wound area was calculated. **G**, **H** Masson’s trichome staining of wound sections in control, exosome^Old^ and exosome^Young^ groups (scale bar: 200 μm). **I** Immunofluorescence staining for α-SMA was used to examine the density of myofibroblasts (scale bar: 50 μm). **J**, **K** α-SMA and Col1 mRNA levels were analyzed. ^*^P < 0.05 versus exosome^Young^ in one-way analysis of variance (n = 6)

The phenotypic switch of fibroblasts to myofibroblasts is important for successful primary wound closure in young mice. We tested whether this effect could also be achieved in aged mice by administration of exosomes derived from fibroblasts isolated from the wound edge of young mice (exosome^Young^). Exosome^Young^ and exosome^Old^ were applied directly to an excisional skin wound in aged mice via subcutaneous injection around the wound (Fig. 2A). The exosomes exhibited a round morphology and size of 50–100 nm, according to TEM and NTA. Moreover, expression of the exosome markers ALIX, CD9 and TSG101 were confirmed by western blot (Fig. 2B–D).

Then, using the aged-mouse wound model, we studied the effects of exosome^Young^ on wound healing and fibroblast transition. We found that wounds treated with exosome^Young^ showed significantly accelerated wound closure compared with the rate of control-treated wounds, whereas wounds treated with exosome^Old^ showed minimal difference (Fig. 2E, F). Additionally, we found that exosome^Young^ alleviated the defect in collagen deposition in wound healing caused by aging, while exosome^Old^ did not affect deposition (Fig. 2G, H). Furthermore, when we analyzed FMT in the wound-edge skin, we observed more α-SMA + cells in the exosome^Young^-treated group (Fig. 2I), which was accompanied by elevated expression of α-SMA and Col1 (Fig. 2J, K).

### Identification of major mechanisms associated with exosome ^Young^ -guided wound healing and FMT

**Fig. 3:**
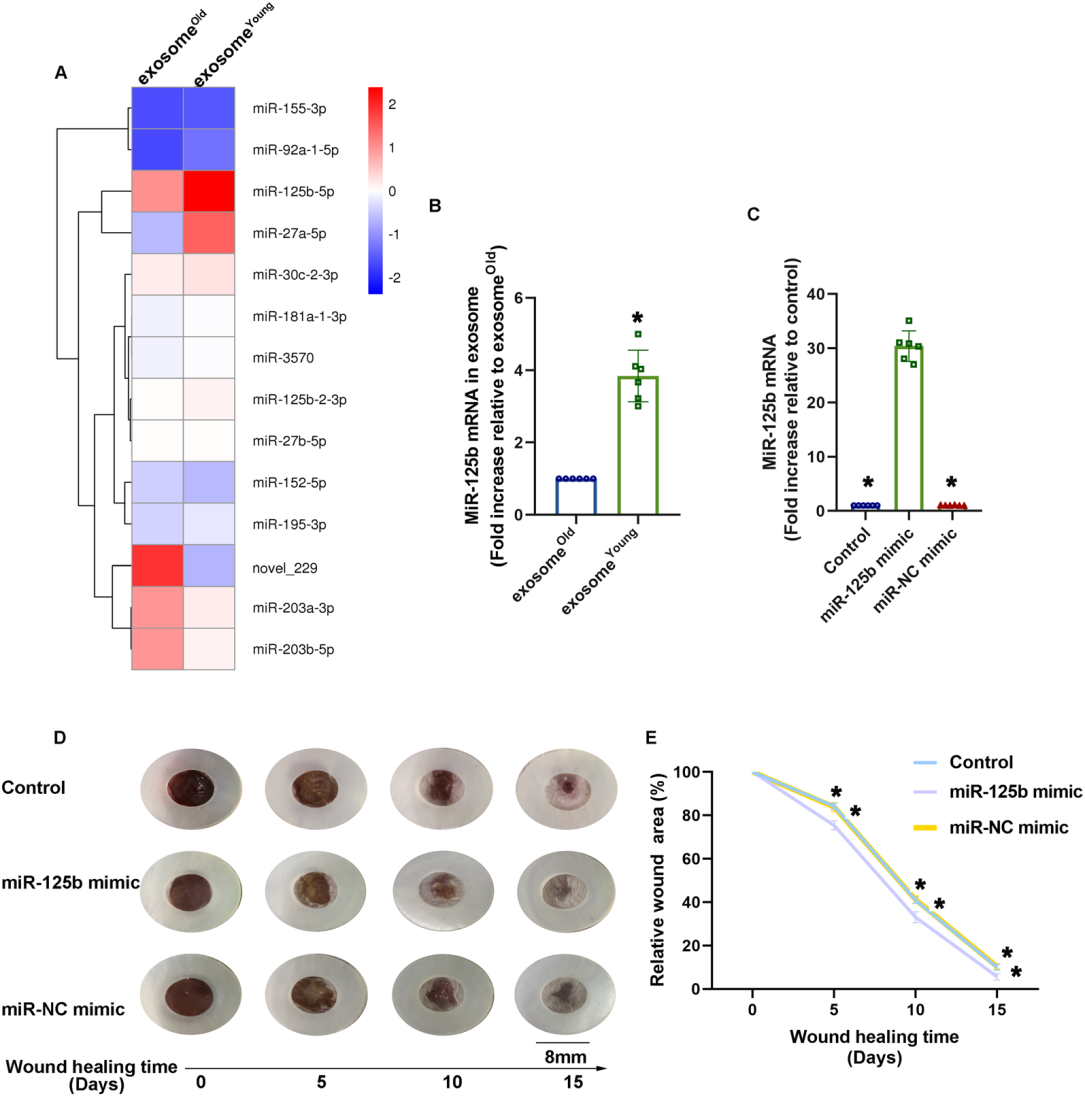
MiR-125b is involved in exosome^Young^ -mediated wound healing.**A** Heat map of miRs differentially expressed in exosome^Old^ and exosome^Young^. **B** MiR-125b expression was validated by qRT-PCR in exosome^Old^ and exosome^Young^. ^*^P < 0.05 versus exosome^Old^ in paired t-test (n = 6). **C** MiR-125b expression was validated by qRT-PCR after transfection of miR-125 mimic, or miR mimic negative control (NC) intradermally into the wound-edges. **D** Representative images of wound closure after local transfection of miR-125 mimic, or miR-NC mimic. **E** Relative wound area was calculated. ^*^P < 0.05 versus miR-125b mimic in one-way analysis of variance (n = 6)

To identify the molecular mechanisms underlying the exosome^Young^ repair effect, exosome^Young^ and exosome^Old^ were collected and analyzed by microarray. Interestingly, the fibrotic-related miR, miR-125b, was increased in exosome^Young^ (Fig. 3A) and confirmed by qRT-PCR (Fig. 3B). Due to the pro-healing effect of exosome^Young^, and the miR-125b was enriched in exosome^Young^, we hypothesized that the replenishment of miR-125b might be a therapeutic approach to promote healing. We injected miR-125b mimics intradermally into the wound-edges immediately following an injury. This treatment specifically and efficiently increased the levels of miR-125b in the wounds (Fig. 3C). Also, we observed significantly accelerated wound closure (Fig. 3D, E).

**Fig. 4:**
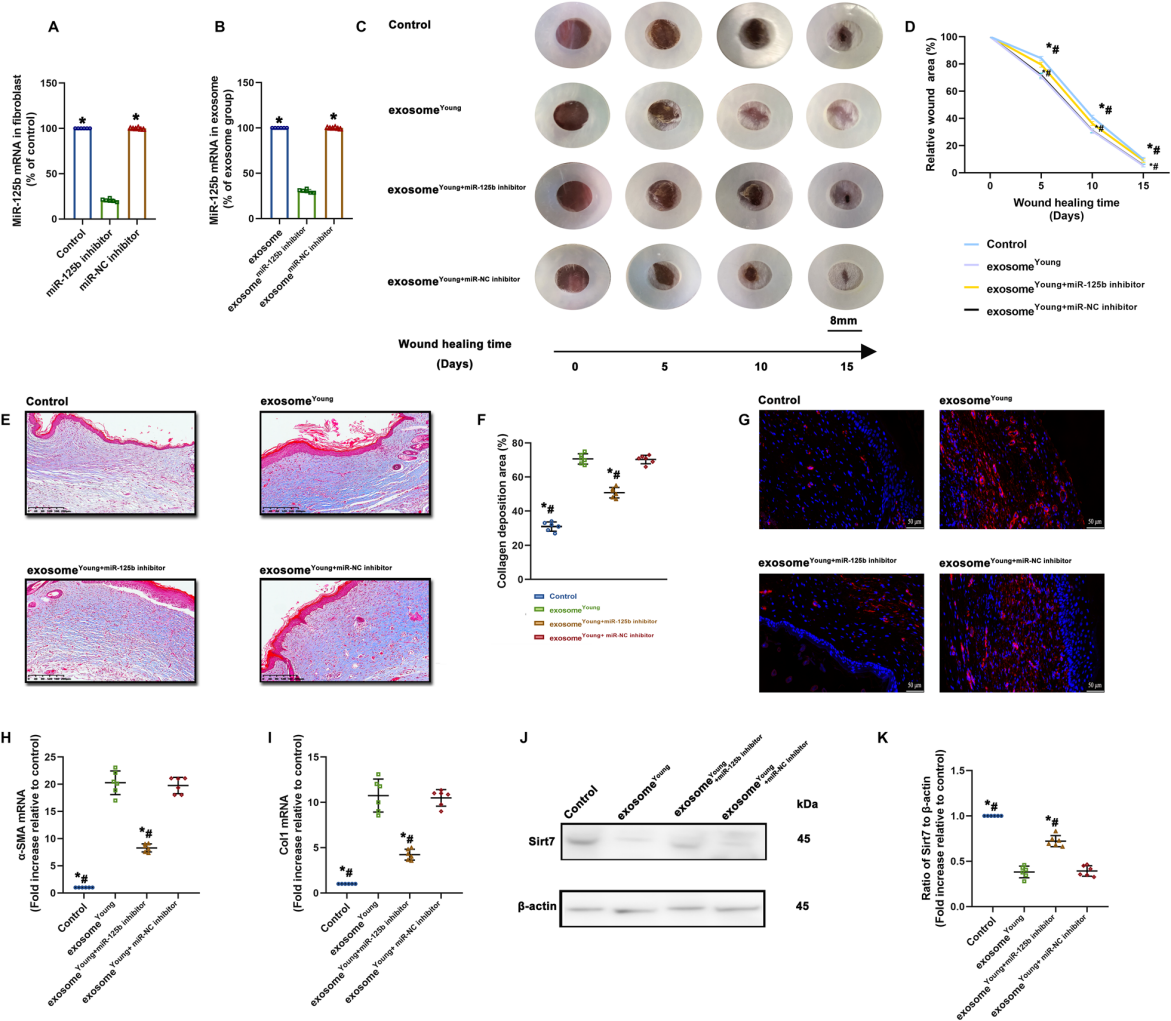
Identification of major mechanisms associated with exosome ^Young^ -guided wound healing and FMT. **A** MiR-125b expression was validated by qRT-PCR in fibroblasts after transfection of miR-125b inhibitor, or miR inhibitor negative control (NC). ^*^P < 0.05 versus miR-125b inhibitor in repeated measures analysis of variance (n = 6). **B** MiR-125b expression was validated by qRT-PCR in exosomes after inhibiting miR-125b in fibroblasts. ^*^P < 0.05 versus exosome^miR−125b inhibitor^ in repeated measures analysis of variance (n = 6). **C** Representative images of wound closure after local injection of saline (Control), exosome^Young^, exosome^Young+miR−125b inhibitor^ or exosome^Young+miR−NC inhibitor^. **D** Relative wound area was calculated. **E**, **F** Masson’s trichome staining of wound sections in control, exosome^Young^, exosomes^Young+miR−125b inhibitor^ or exosome^Young+miR−NC inhibitor^ groups (scale bar: 200 μm). **G** Immunofluorescence staining for α-SMA was used to examine the density of myofibroblasts (scale bar: 50 μm). (H and I) α-SMA and Col1 mRNA levels were analyzed. **J**, **K** Western blot analysis of Sirt7 and β-actin protein levels. *P < 0.05 versus exosome^Young^; ^#^*P* < 0.05 versus exosome^Young+miR−NC inhibitor^ in one-way analysis of variance (n = 6)

Since the exosome^Young^ healing effects in aged mice may be associated with delivery of miR-125b, we investigated the potential signaling pathways that could impact fibroblast function during repair. We inhibited the expression of miR-125b in fibroblasts using an miR-125b inhibitor, then exosomes (exosome^Young+miR−125 inhibitor^) derived from the above cells were collected. QRT-PCR results revealed that expression of miR-125b not only decreased in the fibroblasts, but also in exosome^Young+miR−125 inhibitor^ (Fig. 4A, B). As a result, the wounds treated with exosome^Young^ displayed pronounced wound-healing efficacy throughout the entire wound healing process (Fig. 4C, D), with more ECM deposition (Fig. 4E, F). Also, a greater percentage of α-SMA + cells were observed in the exosome^Young^-treated group (Fig. 4G), with elevated expression of α-SMA and Col1 (Fig. 4H, I). However, these healing and pro-fibrotic effects were abolished by miR-125b inhibition in the fibroblasts before isolation of exosome^Young^, suggesting that exosome^Young^ exerted their protective effect through miR-125b transfer. We next examined the expression of Sirt7, an important target of miR-125b. In contrast to controls, expression of Sirt7 decreased more robustly in the exosome^Young^-treated group; however, recovery was observed after miR-125b was inhibited in the exosomes (Fig. 4J, K).

### Exosome ^Young^ activated fibroblast migration and increased fibrosis

**Fig. 5:**
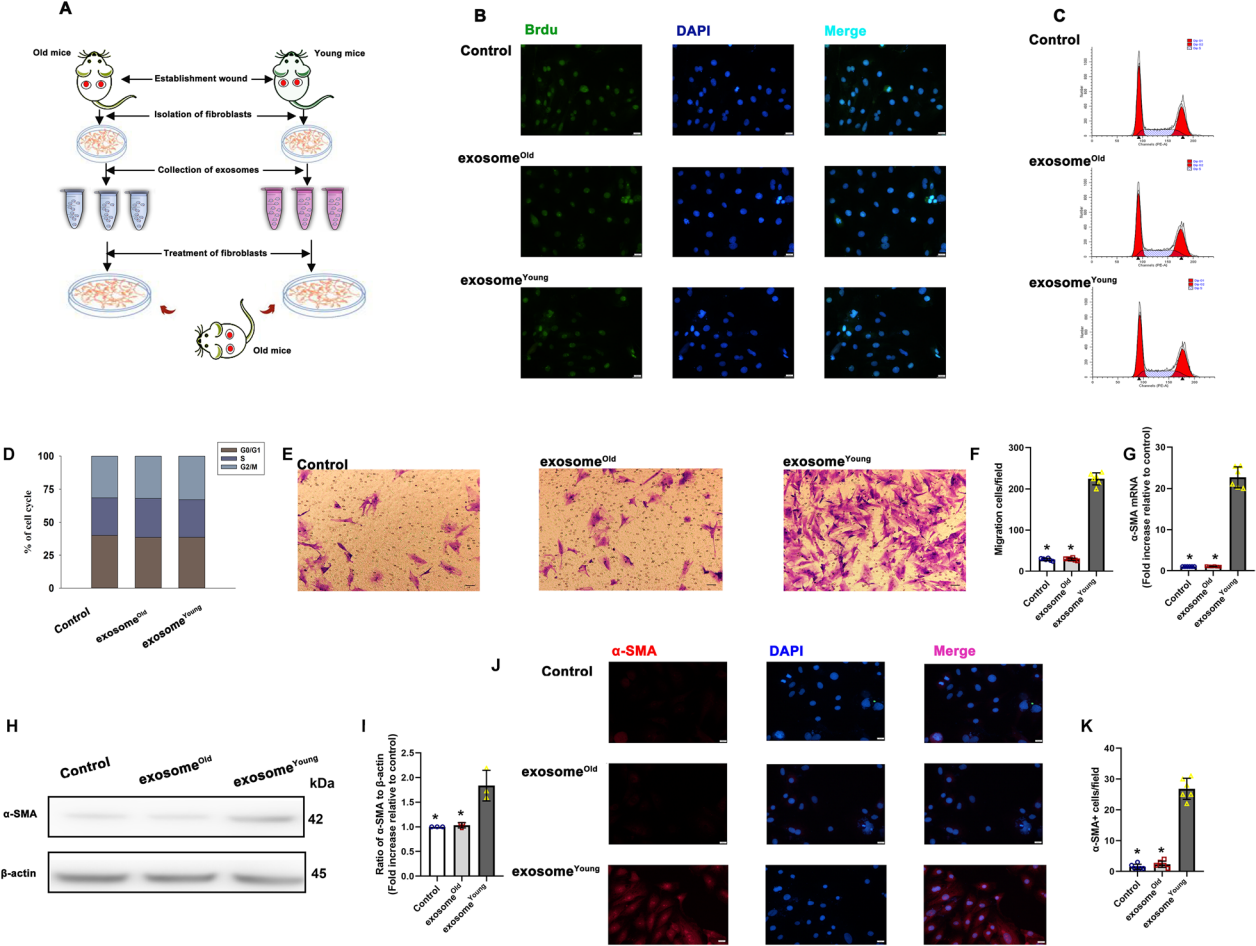
Exosome^Young^ activated fibroblast^Old^ migration and induced fibrosis. **A** Schematic illustration of fibroblast^Old^ treatment. **B** Cellular proliferation was evaluated by Brdu staining. **C**, **D **Cell cycle distribution was analyzed. **E** Images of migrated fibroblast^Old^ using Transwell migration assays. **F** Data are presented as the number of migrated cells. **G, I** Expression of α-SMA was determined using qRT-PCR and western blot analysis. **J** Representative images of immunofluorescence staining of α-SMA in fibroblast^Old^. **K** Quantification of α-SMA-positive cells. ^*^P < 0.05 versus exosome^Young^ in repeated measures analysis of variance (n = 6)

During the wound healing process, proliferation, migration and transition of fibroblasts induce wound repair [29]. Herein, we preferentially evaluated the *in vitro* wound-healing effects of exosome^Young^ on the wound-healing activity of fibroblasts isolated from aged mice wound edges (fibroblast^Old^) (Fig. 5A). First, the pattern of fibroblast proliferation was examined by performing Brdu staining and cell cycle analysis. Interestingly, no acceleration of proliferation was observed when fibroblast^Old^ were exposed to exosome^Young^ (Fig. 5B). Meanwhile, the administration of exosome^Young^ did not affect the cell cycle (Fig. 5C, D). As expected, the fibroblasts co-cultured with exosome^Young^ showed improved migration, while the exosome^Old^-exposed cells were not significantly different from the control group (Fig. 5E, F). During the mid-phase of wound healing, FMT has a great effect on ECM remodeling [30]. To confirm the inducing effects of exosome^Young^ on FMT, expression of α-SMA was evaluated. Results of qRT-PCR and western blot indicated that exosome^Young^ induced more expression of α-SMA (Fig. 5G, I), at meanwhile, immunofluorescence assays revealed more α-SMA + cells in the exosome^Young^-treated group compared with either exosome^Old^ or control group cells (Fig. 5J, K).

### Exosome ^Young^ promoted migration and FMT by delivering miR-125b

**Fig. 6:**
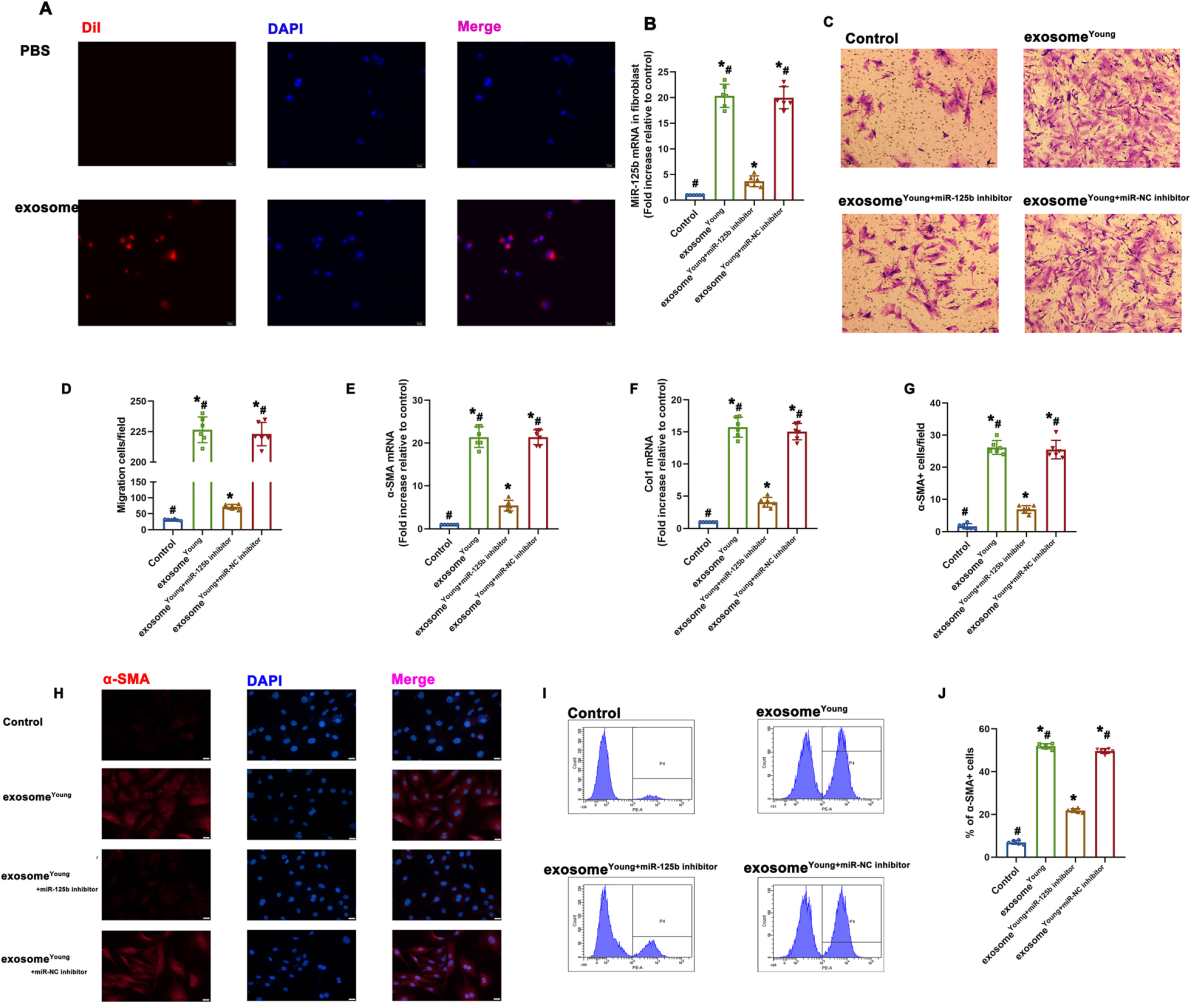
Exosome^Young^ promoted migration and FMT by delivering miR-125b. **A** DiI-labeled exosomes were engulfed by fibroblasts as shown. Scale bar: 20 μm. **B** MiR-125b mRNA in fibroblast^Old^ was examined using qRT-PCR. ^*^P < 0.05 versus Control; ^#^*P* < 0.05 versus exosome^Young+miR−125b inhibitor^ in repeated measures analysis of variance (n = 6). **C** Images of migrated fibroblasts^Old^ using Transwell migration assays. **D** Data are presented as the number of migrated cells. **E**, **F** The mRNA levels of the pro-fibrotic genes α-SMA and Col1 were analyzed using qRT-PCR. **G** Quantification of α-SMA-positive cells. **H** Representative images of immunofluorescence staining of α-SMA. **I** FACS plots detailing the gating strategy to define SMA-positive subpopulations. **J** Quantification of the relative abundance of SMA-positive cells. ^*^P < 0.05 versus Control; ^#^*P* < 0.05 versus exosome^Young+miR−125b inhibitor^ in repeated measures analysis of variance (n = 6)

Since the delivery of miR-125b increased wound-healing effects in vivo, we investigated the direct effects of miR-125b on old fibroblast. First, higher level of miR-125b after transfected with correspondent miRNA mimic was observed in old fibroblast. (Additional file 1: Fig.S1A). Then the effects of miR-125b on fibroblast migration and FMT were measured. The results suggested that miR-125b mimic not only accelerated cell migration (Additional file 1: Fig.S1B, C), but also facilitated the transition of fibroblast to myofibroblast, with higher expression of α-SMA + and Col1 (Additional file 1: Fig.S1D, E), and a greater percentage of α-SMA + cells (Additional file 1: Fig.S1F, G). These results indicated that miR-125b might be the key regulatory cargo contained in exosome^Young^ accounting for the modulation of fibroblast migration and FMT, so we sought to explore whether exosomal miR-125b could induce fibroblast^Old^ migration and FMT. Exosome^Young^ were labeled with DiI to confirm whether this miR could be transferred to fibroblast^Old^ through exosomes. Exosome uptake by fibroblast^Old^ was observed (Fig. 6A). In addition, expression of miR-125b in fibroblast^Old^ was decreased in the exosome^Young+miR−125 inhibitor^-treated group, suggesting that inhibition of miR-125b impaired miR delivery (Fig. 6B). Transwell migration assays revealed that exosome^Young^ increased the migration of fibroblast^Old^. In contrast, miR-125b inhibition led to a decrease in migration (Fig. 6C, D). To further analyze the pro-fibrotic effect of exosome^Young^, we examined the expression of pro-fibrotic genes α-SMA and Col1. Elevated expression of these pro-fibrotic genes was observed in the exosome^Young^ group, but was reversed in the exosome^Young+miR−125 inhibitor^ group (Fig. 6E, F). Meanwhile, results from immunofluorescence staining and FACS suggested that a greater percentage of α-SMA + cells were induced when exposed to exosome^Young^, while α-SMA + cells decreased in the exosome^Young+miR−125 inhibitor^ group (Fig. 6G–J).

### MiR-125b/Sirt7 signaling activated migration and FMT of wound-bed fibroblasts^Old^

**Fig. 7:**
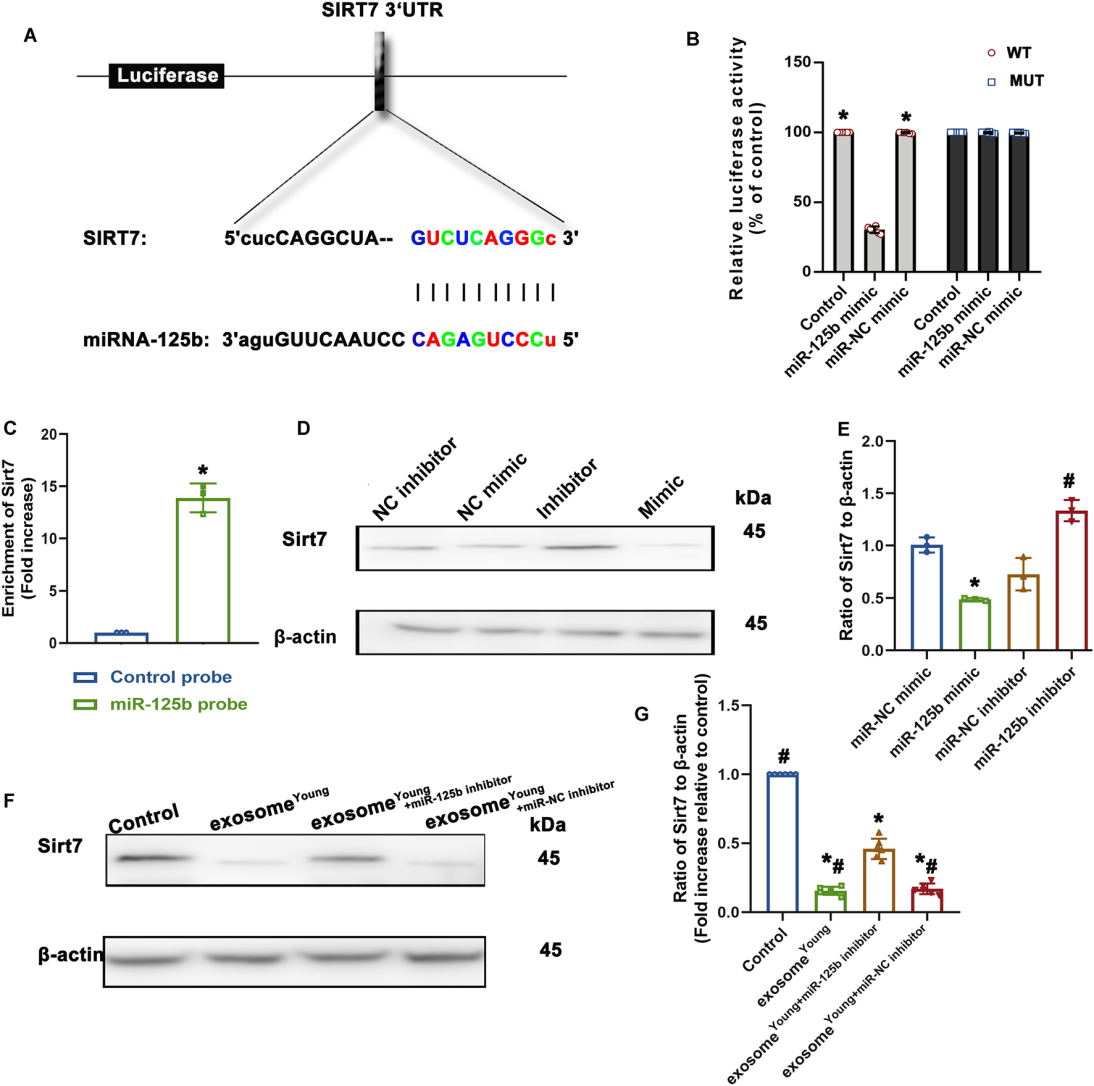
MiR-125b directly targeted Sirt7. **A** Predicted binding sites between miR-125b and the Sirt7 3’-UTR. **B** Dual-luciferase assays were performed in fibroblasts after co-transfection with SIRT7 3’-UTR wild-type (WT) or mutant (MUT) plasmids, and miR-125b mimics or miR-NC mimic. ^*^P < 0.05 versus miR-125b mimic in repeated measures analysis of variance (n = 6) in the WT group. **C** QRT-PCR for Sirt7 treated with RNA pull down assay. ^*^P < 0.05 versus Control probe in paired t-test (n = 3). **D**, **E** Western blot was used to analyze protein level of fibroblasts treated with miR-125b mimic, miR-NC mimic, miR-125b inhibitor, miR-NC inhibitor respectively. ^*^P < 0.05 versus miR-NC mimic; ^#^*P* < 0.05 versus miR-NC inhibitor in paired t-test (n = 3). **F**, **G** Western blot analysis of Sirt7 and β-actin protein levels in fibroblast^Old^ treated with exosome^Young^, exosome^Young+miR−125b inhibitor^ or exosome^Young+miR−NC inhibitor^. Untreated fibroblast^Old^ were used as control. ^*^P < 0.05 versus Control; ^#^*P* < 0.05 versus exosomes^Young+miR−125b inhibitor^ in repeated measures analysis of variance (n = 6)

Since miRs affect the regulation of their target genes, TargetScan was applied to search for the target gene of miR-125b. Sirt7 was identified as a possible target of miR-125b because miR-125b suppresses Sirt7 and thus further activates TGF-β signaling, promoting cellular migration [31]. Dual-luciferase gene reporter assays were used to confirm the hypothesis of a putative binding site for miR-125b in Sirt7 (Fig. 7A). Weakened relative luciferase activity was observed in the wild-type Sirt7 + miR-125b mimic group (Fig. 7B). To determine whether Sirt7 was the target of miR-125b, biotinylated miR-125b was used as a probe to pull down its binding partners in vitro, and qRT-PCR was applied to quantify Sirt7 captured by the miR-125b probe. The pull-down assay showed more cellular Sirt7 could be captured than that of nonspecific control probe (Fig. 7C). Furthermore, fibroblasts were treated with miR-125b mimic, miR-NC mimic, miR-125b inhibitor, miR-NC inhibitor respectively. Overexpression of miR-125b was sufficient to inhibit Sirt7, in contrast, simultaneous inhibiting miR-125b increased Sirt7 (Fig. 7D, E). Exosome^Young^-treated fibroblast^Old^ were then used to investigate the inhibitory effect of exosomal-delivered miR-125b on Sirt7 expression. As expected, exosome^Young^ significantly inhibited Sirt7 expression in fibroblast^Old^, while the inhibition of miR-125b in exosomes rescued the expression of Sirt7 (Fig. 7F, G). Taken together these data suggest that the miR-125b acts upstream of Sirt7.

To better understand how miR-125b/Sirt7 modulates fibroblast, we focused on Sirt7, the target gene of miR-125b. Small interfering-RNA was applied to explore the effect of Sirt7 on fibroblast migration and FMT. SiRNA knockdown of siRNA-Sirt7 induced a decrease in expression of Sirt7 (Additional file 2: Fig.S2A–C). Sirt7 knockdown induced increase in cell migration (Additional file 2: Fig.S2D, E); expression of α-SMA + and Col1 (Additional file 2: Fig.S2F, G), and a greater percentage of α-SMA + cells (Additional file 2: Fig.S2H, I). These data imply that reduction in Sirt7 can induce the migration and FMT of fibroblast^Old^.

**Fig. 8:**
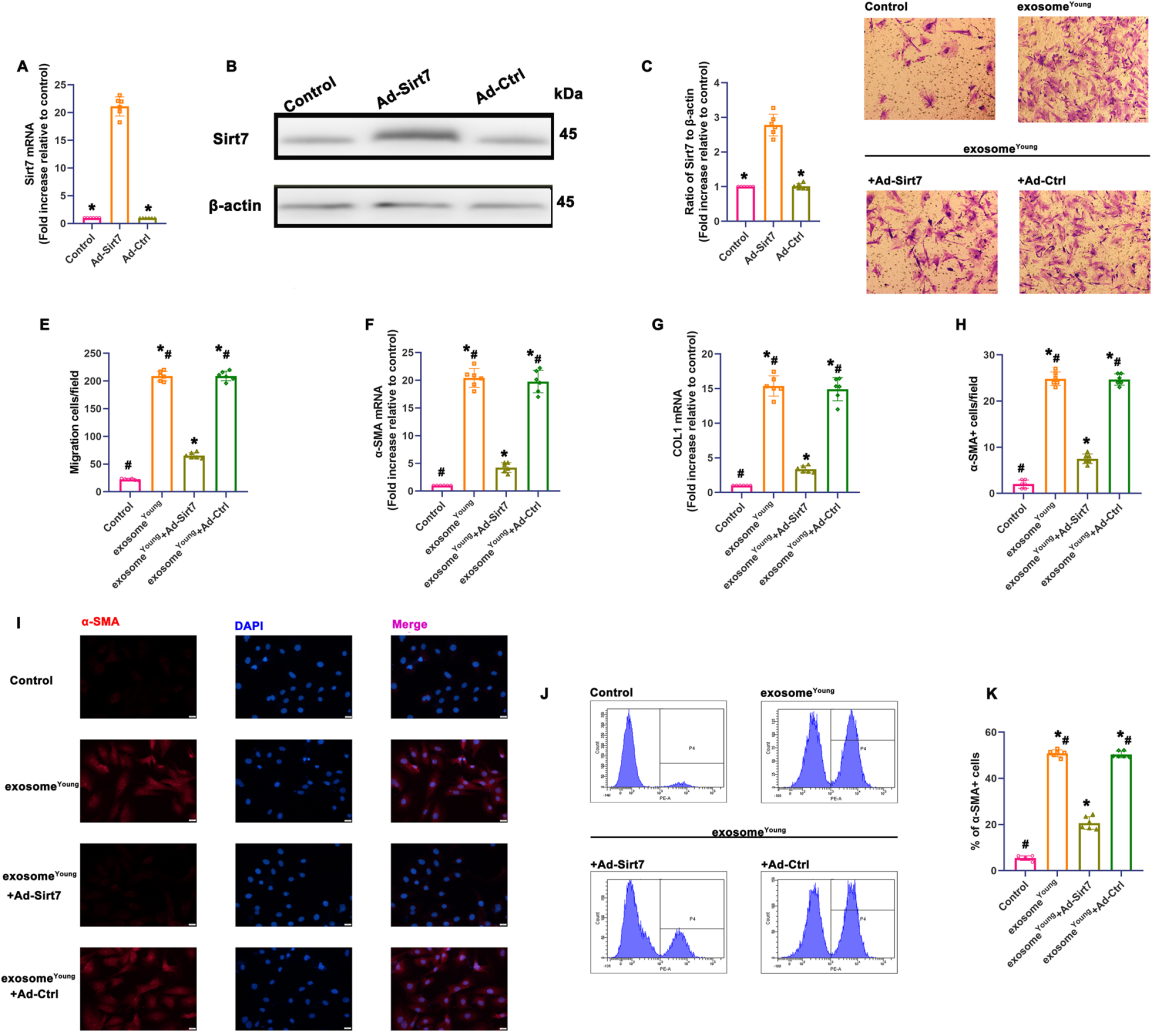
Mir-125b/Sirt7 signaling activated migration and FMT of wound-bed fibroblasts ^Old^. **A**–**C** Fibroblasts^Old^ were transfected with Ad-Sirt7 or Ad-Ctrl as control. Transfection efficiency was determined using qRT-PCR **A** and western blot analysis (**B**, **C**). ^*^P < 0.05 versus Ad-Sirt7 in repeated measures analysis of variance (n = 6). **D** Images of migrated fibroblasts^Old^ using Transwell migration assays. (E) Data are presented as the number of migrated cells. **F**, **G** Pro-fibrotic genes α-SMA and Col1 mRNA levels were analyzed using qRT-PCR. **H** Quantification of α-SMA-positive cells. **I** Representative images of the immunofluorescence staining of α-SMA. **J** FACS plots detailing the gating strategy to define SMA-positive subpopulations. **K** Quantification of the relative abundance of SMA-positive cells. ^*^P < 0.05 versus Control; ^*#*^*P* < 0.05 versus exosome^Young^ + Ad-Sirt7 in repeated measures analysis of variance (n = 6)

To determine whether repair-related exosomal miR-125b arose from Sirt7 inhibition, we performed Ad-Sirt7 transfection to induce Sirt7 overexpression in fibroblast^Old^. We measured the overexpression efficiency using qRT-PCR (Fig. 8A) and western blots (Fig. 8B, C). Increased expression of Sirt7 led to a significant decrease in migration (Fig. 8D, E). Comparing expression of fibrotic genes α-SMA and Col1 in exosome^Young^-treated cells and exosome^Young^+Ad-Sirt7-transfected fibroblast^Old^ confirmed significant inhibition of these genes by Sirt7 overexpression (Fig. 8F, G). We also observed a decreased percentage of α-SMA + cells after overexpression of Sirt7 (Fig. 8H–K). Therefore, we ruled out the possibility that inhibition of Sirt7 induced by miR-125b contributed to the pro-fibrotic effect of exosome^Young^.

## Discussion

Numerous studies have shown that wound healing is delayed in aged tissues [32, 33]. With the dramatic increase in the size of the elderly population over 65 years of age, the medical, economic, and social burdens posed by nonhealing wounds are accelerating [34]. Wound healing is a complex biological process which requires the interaction of diverse cell types and cellular activities [10]. These cellular activities, including migration, proliferation, differentiation, and synthesis of ECM proteins, are tightly regulated and are crucial for optimal repair, which must be orchestrated in a spatiotemporal manner to achieve proper FMT and ECM deposition [35].

Age-related alterations have been described in all three stages of the healing process, especially in the second stage, with delays in new tissue formation [10, 36]. During this phase of new tissue formation, fibroblasts stimulated by TGF-β1 migrate to the wound bed, differentiate into myofibroblasts, and ultimately synthesize ECM components [37]. However, this phase may be delayed or even impaired during aging. Fibroblasts from aged donors also exhibit depressed migratory activity. Moreover, there is an age-related decrease in responsiveness to TGF-β1, resulting in a decreasing number of myofibroblasts [38]. The molecular underpinnings explaining why such delays in wound healing occur during aging were examined in our research. In our study, both deficiencies in α-SMA + fibroblasts and ECM deposition contributed to impaired healing in aged skin. We detected reduced FMT and collagen formation at the wound sites of aged versus young animals, in agreement with a previous study suggesting that age-related defects in repair were associated with reduced myofibroblasts and dysfunctional ECM deposition [11]. Degradation of ECM, one of the classical functions of the MMP family, was also abnormally activated in aged skin, resulting in delayed healing [39]. Here in our study, when aged and young animals were placed into equivalent environments, gene expression of multiple MMPs including MMP3, MMP9 and MMP12 was increased, suggesting that the ability of aged fibroblasts to break down ECM faster than younger fibroblasts may contribute to the impaired healing process. All the above features pointing to age-related defects in wound healing were accompanied by age-related perturbations in fibroblast behavior and ECM deposition during wound-repair.

Recent evidence has implicated cell-systemic environmental interactions which affect several facets of wound repair in multiple tissue types [40]. Importantly, migrating fibroblasts localize around newly formed blood vessels, allowing for potential communication with the systemic environment [34]. Therefore, the possibility arises that age-related physiological changes which negatively affect wound healing could be alleviated by delivery of extracellular vesicles. Exosomes, cell-derived extracellular vesicles with a diameter 30–150 nm, have been shown to mediate cell-systemic environment communication by transferring a particular mixture of proteins, lipids, miRs and long noncoding RNAs [41]. In this regard, the exosome-mediated cell–systemic environmental interaction has been described as a potential way to interact with neighboring cells, thus leading to regenerative effects in aged tissue [42]. Exosomes derived from kinds of stem cells, such as human neural stem cells, showed regenerative potentials [43]. When coupled with the age-induced wound-healing impairment resulting from inefficient FMT of fibroblasts, our data demonstrate that exosomes derived from young fibroblasts located at the wound bed is critical for wound restoration. Indeed, our findings show that exosome^Young^ can enhance both fibroblasts^Old^ migration and FMT, thus reducing the wound healing impairment in aged mice. When placed into equivalent environments *in vitro*, aged basal fibroblasts isolated from the wound edge appear to be more resistant to differentiation into myofibroblast, as judged by the markedly reduced transcriptional activity of genes involved in FMT such as α-SMA and Col1. By contrast, the exosome^Young^-treated group not only increase migration but also induced FMT when compared with control counterparts. Thus, the efficient exchange of cellular components through exosomes can inform their applied use in designing exosome-based therapeutics [44].

Finally, we traced the target that could induce the promotional effect of exosome^Young^ on fibroblasts. MiRs, which have emerged as key regulators of physical homeostasis during aging [45], have been examined during wound healing [46]. As a fundamental function of exosomes, modification of gene expression can be carried out through the transfer of miRs [47]. Comparing the transcriptome of exosomes from fibroblasts isolated from young versus aged donors revealed increased expression of miR-125b. Our in vivo and vitro results indicated that miR-125b served as the key regulatory cargo contained in exosome^Young^ accounting for the wound healing. At meanwhile, in our study, we detected a high level of miR-125b in the exosomes, as well as in exosome-treated fibroblasts. Moreover, DiI staining results revealed that exosomes could be engulfed by fibroblasts, indicating that miR-125b can be transferred from exosomes to recipient cells, consistent with a previous study showing that miR-125b delivery by extracellular vesicles conferred fibroblast activation [48]. Importantly, miR-125b is necessary and sufficient for the induction of the FMT. Our data also underscore a mechanistic role for exosome-mediated miR-125b/Sirt7 signaling in driving fibroblast migration and FMT during the youthful wound response, and reveal an overall function for this circuitry in aged mice. We traced the elusive target that affected exosomal-transfer behavior of miR-125b to Sirt7. Exosomal delivery of miRs has been shown repress the expression of Sirt7 to activate the TGF-β1 pathway, resulting ECM deposition [49]. In our study, we observed a similar phenomenon in which exosomal-delivered miR-125b induced activation of the TGF-β1 pathway, which was confirmed by elevation of fibrosis-related gene expression and induction of FMT. Further study revealed that ectopic Sirt7 overexpression impaired the activation of this pathway. Sirt7, a sirtuin family member implicated in aging and disease, is a regulator of metabolism and stress responses [50]. With respect to aging, transcriptional suppression of Sirt7 activates TGF-β signaling, thus promoting cell survival and migration [31]. The number of α-SMA + fibroblasts in our study increased as Sirt7 abundance decreased. Thus, the interaction between the miR-125b/Sirt7 pathway and fibroblasts may provide a therapeutic target for age-related deficiency in wound healing.

## Conclusions

In summary, this work provides critical insight into the significance of exosome^Young^ in the resolution of reduced myofibroblasts and dysfunctional ECM deposition during aged wound closure, acting via exosomal miR-125b packaging and phenotypic inhibition of Sirt7. This advance in our understanding of aged wound healing unveils heretofore unknown therapeutic targets that may be exploited to design effective wound-healing strategies in older people.

## Materials and methods

### Animals

Eight-week-old and 18-month-old male Balb/c mice were maintained in accordance with the guidelines published by the US National Institutes of Health. This study was conducted in compliance with the Guide for the Care and Use of Laboratory Animals published by the National Academy Press (NIH, revised in 1996). All study procedures were approved by the Institutional Animal Care and Use Committee of Wenzhou Medical University.

### Wound healing model and treatment

The mice were anesthetized by 2.5% of isoflurane. The hair on the back of mice was removed by electric shaver and sterilized using 70% alcohol. The 8 mm skin wounds were made using biopsy punch. 100 µg per 100 µL exosomes were treated from day 1 to day 5. The diameter of each wound (day 0, 5, 10, and 15) was measured using Photoshop to calculate the percentage of wound closure.

To induce locally expression of miR-125b, 1 nmol miR-125b mimic or negative control (ThermoFisher Scientific) in a total volume of 100 µl for each wound was injected intradermally into the wound-edges of mice according to manufacturer’s instruction, as previously reported [51].

### Quantitative reverse transcription–polymerase chain reaction (qRT-PCR)

RNA was isolated using TRIzol reagent (Invitrogen, Carlsbad, USA), and cDNA was synthesized using an Improm II reverse transcription kit (Promega, WI, USA). QRT-PCR was performed with SYBR Green to detect mRNA levels. The mRNA levels were calculated relative to the control Gapdh (for mRNA) or U6 (for miRNA) using the 2^−ΔΔCq^ method.

### Histological analysis

Tissue samples were fixed in 4% paraformaldehyde and 3 μm sections were cut from paraffin embedded blocks. Sections were stained with standard Masson’s trichrome.

### Immunofluorescence staining

Immunofluorescent staining was performed with paraffin-embedded tissues and 4% PFA fixed fibroblasts. Following primary antibody (α- SMA; ab124964; 1:50) incubation, slides were washed with 1X PBS and incubated with Alexa Fluor 594 secondary for one hour at room temperature. Sections were counterstained with DAPI mounting medium and analyzed. Fluorescence was detected under a microscope.

### Isolation and characterization of exosomes

The exosomes were isolated and purified from the supernatants of fibroblasts isolated from wound edge of aged and young mice, cultured in EV free media. Briefly, following initial centrifugation for 30 min at 3000 × *g*, cells and other debris were removed and the supernatant was harvested and centrifuged at 10,000 × *g* for 30 min to remove microvesicles larger than exosomes. The supernatant was finally centrifuged at 110,000 × *g* for 70 min. The isolation process was performed at 4 °C, and the exosomes were resuspended in PBS and stored at − 80 °C.

The morphology of exosomes was observed by transmission electron microscopy (TEM, JEM-1400plus). The size was determined by nanoparticle tracking analysis (NTA). Western blot analysis was used for detecting exosomal markers ALIX, CD9, and TSG101, as previously reported [52].

### MiRNA microarray analysis

MiRNA enrichment procedure were performed with the mirVana miRNA Isolation Kit (Ambion). RNA concentration was quantified using the Nano Drop spectrophotometer and the RNA integrity was evaluated. Agilent Mouse miRNA Microarray Kit was used as a miRNA microarray chip for hybridization. The microarray images were scanned with the Agilent microarray scanner.

### Dermal fibroblast isolation

Euthanize mice were shaved and depilated the dorsal skin. Immediately harvest dorsal mouse skin using sterile dissecting scissors. Mince the skin to 2–3 mm pieces. Then digested with collagenase IV at a concentration of 1 mg/ml in DMEM, incubating at 37 °C for 1 h. Pipette the sample through a 100 μm filter into conical tube, neutralizing with 10% FBS DMEM. Centrifuge at 300 g for 8 min at 4 °C. Then remove supernatant and the upper fat layer. Resuspend the pellets in 20 ml 10% FBS DMEM. Pass the cell/DMEM suspension through a 70 μm filter. Rinse the filter with 10 ml 10% FBS DMEM and centrifuge the filtered suspension at 300 g for 8 min at 4 °C. Re-suspend the pellets and an equal volume of FACS buffer was added. Then, FACS analysis was performed, sorting for viability cells, as previously reported [53].

### Cell proliferation assay

Cellular proliferation was analyzed with BrdU staining as previously reported [54]. Briefly, 1 × 10^4^ cells, culturing on coverslips in 24-well plates were incubated with 10 µg/ml BrdU, washed with 1 × PBS, then fixed with 4% paraformaldehyde. After blocked with 5% goat serum for 2 h, the cells were incubated with a primary antibody against BrdU for 1 h, followed by Alexa Fluor® 488 secondary antibody. DAPI was used for nuclear staining.

.

### Cell cycle assay

Cold anhydrous ethanol (70%) was employed to fix the cells. Then, the cells were treated with propidium iodide (Sigma, MO, USA) and RNase A. A flow cytometer equipped with CellQuest software was used to detect the cell cycle distribution.

### Transwell migration assay

The 8 μm pore size Transwell chambers (Corning, NY, USA) were applied to determine the cellular migration, as previously reported [55]. Five random fields were counted per chamber using an inverted microscope (Olympus, Tokyo, Japan).

### MiR-125b modulation in vitro

The fibroblasts were seeded into six-well plates at a density of 1 × 10^5^ cells per well and incubated for 12 h. To induce the inhibition or overexpression of miR-125b, the cells were transfected with miR-125b inhibitor or negative control (NC) inhibitor (Invitrogen); miR-125b mimic or negative control (NC) mimic (Invitrogen) using X-treme transfection reagent (Roche Applied Science, Penzberg, Germany), according to the manufacturer’s protocol. The cells were harvested for further analysis 48 h after transfection, and the transfection efficiency was analyzed using qRT-PCR.

### Dual-luciferase reporter gene assay

StarBase database was applied to search for specific binding regions between the Sirt7 gene sequence and the miR-125b sequence. The WT-Sirt7 and Mut-Sirt7 vectors were constructed, respectively. The reporter vectors, miR-125b mimic plasmid and NC plasmid, were co-transfected into 293 T cells, respectively. After 24 h of transfection, the cells were harvested, lysed, and centrifuged. Luciferase assays were performed using a dual-luciferase reporter assay system (Promega).

### Pull-down assay

A biotin-labeled miR-125b pull-down probe and the negative control probe was synthesized. The binding reactions were performed, as described previously [56]. The levels of pulled-down Sirt7 were analyzed by qRT-PCR.

### Western blot analysis

The cells were harvested, and total protein was extracted using RIPA solution. After normalizing for equal protein concentration, cell lysates were resuspended in SDS sample buffer before separation by SDS-PAGE. Western blots were performed using the following antibodies: ALIX (ab275377; 1:1000); CD9 (ab92726; 1:750); TSG101 (ab125011; 1:1000); Sirt7 (ab259968; 1:1000); α-SMA (ab124964; 1:500); Col1 (CST, #81,375; 1:500); β-actin (ab179467, 1:1000). The membranes were further incubated with IgG-horseradish peroxidase goat anti-rabbit secondary antibody (ab7090: 1:2000) for 2 h at room temperature. The signals were developed by enhanced chemiluminescence (CST, #6883). The stained protein bands were visualized using a Bio-Rad ChemiDoc XRS imaging system and analyzed using Quantity One software.

### Small interfering RNA transfection

Sirt7 expression in fibroblasts was knocked down using small interfering (si)RNAs, with a nontargeting siRNA as a negative control (Invitrogen). The procedures were conducted as described previously [57]. The transfection efficiency was detected using qRT-PCR and western blot analysis.

### Transient transfection

For overexpression of Sirt7 in the fibroblasts, they were transduced with adenoviral Sirt7 (Ad- Sirt7) or adenoviral control (Ad-Ctrl) as described previously [58]. The transfection efficiency was confirmed by qRT-PCR and western blot.

### Flow cytometry

To analyze the FMT in the fibroblast, harvested cells were washed with PBS, then the cells were resuspended in FACS staining buffer. Intracellular flow cytometry using αSMA was performed as previously described [59].

### Statistical analysis

Data were expressed as mean ± standard deviation. Statistical significance of differences among groups was tested by one-way ANOVA or repeated measures ANOVA. Comparisons between two groups were done using Student’s t-test or paired t-test. A value of *P* < 0.05 was considered statistically significant.

## Supplementary Information

**Additional file 1: Fig. S1. **MiR-125b promoted fibroblast migration and FMT. **A** MiR-125b mRNA in fibroblast^Old^ after transfecting miR-125b mimic or miR-NC mimic was examined using qRT-PCR. *P < 0.05 versus miR-125b mimic in repeated measures analysis of variance (n = 3). **B** Images of migrated fibroblasts^Old^ using Transwell migration assays. **C** Data are presented as the number of migrated cells. **D**, **E** The mRNA levels of the pro-fibrotic genes α-SMA and Col1 were analyzed using qRT-PCR. **F** FACS plots detailing the gating strategy to define SMA-positive subpopulations. **G** Quantification of the relative abundance of SMA-positive cells. *P < 0.05 versus miR-125b mimic in repeated measures analysis of variance (n = 3).

**Additional file 2: Fig. S2.** Silencing Sirt7 promoted fibroblast migration and FMT. **A**–**C** QRT-PCR** A** and western blot analysis **B**,**C** tested the siRNA-mediated transfection efficiency. **D** Images of migrated fibroblasts^Old^ using Transwell migration assays.**E** Data are presented as the number of migrated cells. **F**, **G** The mRNA levels of the pro-fibrotic genes α-SMA and Col1 were analyzed using qRT-PCR.**H** FACS plots detailing the gating strategy to define SMA-positive subpopulations. **I** Quantification of the relative abundance of SMA-positive cells. *P < 0.05 versus siRNA-Sirt7 in repeated measures analysis of variance (n = 3).

## Data Availability

All data and materials are available in the manuscript.

## Abbreviations

Ad-Ctrl: Adenoviral control
Ad-Sirt7: Adenoviral Sirt7
Col1: Collagen I
DMEM: Dulbecco’s modified Eagle’s medium
ECM: Extracellular matrix
FACS: Flow cytometric analysis cell scan
FMT: Fibroblast-to-myofibroblast transition
miRs: micro RNAs
MMPs: Multiple matrix metalloproteases
NTA: Nanoparticle tracking analysis
qRT-PCR: Quantitative reverse transcription–polymerase chain reaction
SD: Standard deviation
Sirt7: Sirtuin 7
TEM: Transmission electron microscopy
