# Supplementary figures and images for: Young fibroblast-derived exosomal microRNA-125b transfers beneficial effects on aged cutaneous wound healing
Source: J Nanobiotechnology. 2022 Mar 19;20:144. doi: 10.1186/s12951-022-01348-2 (PMC9744129; doi:10.1186/s12951-022-01348-2)

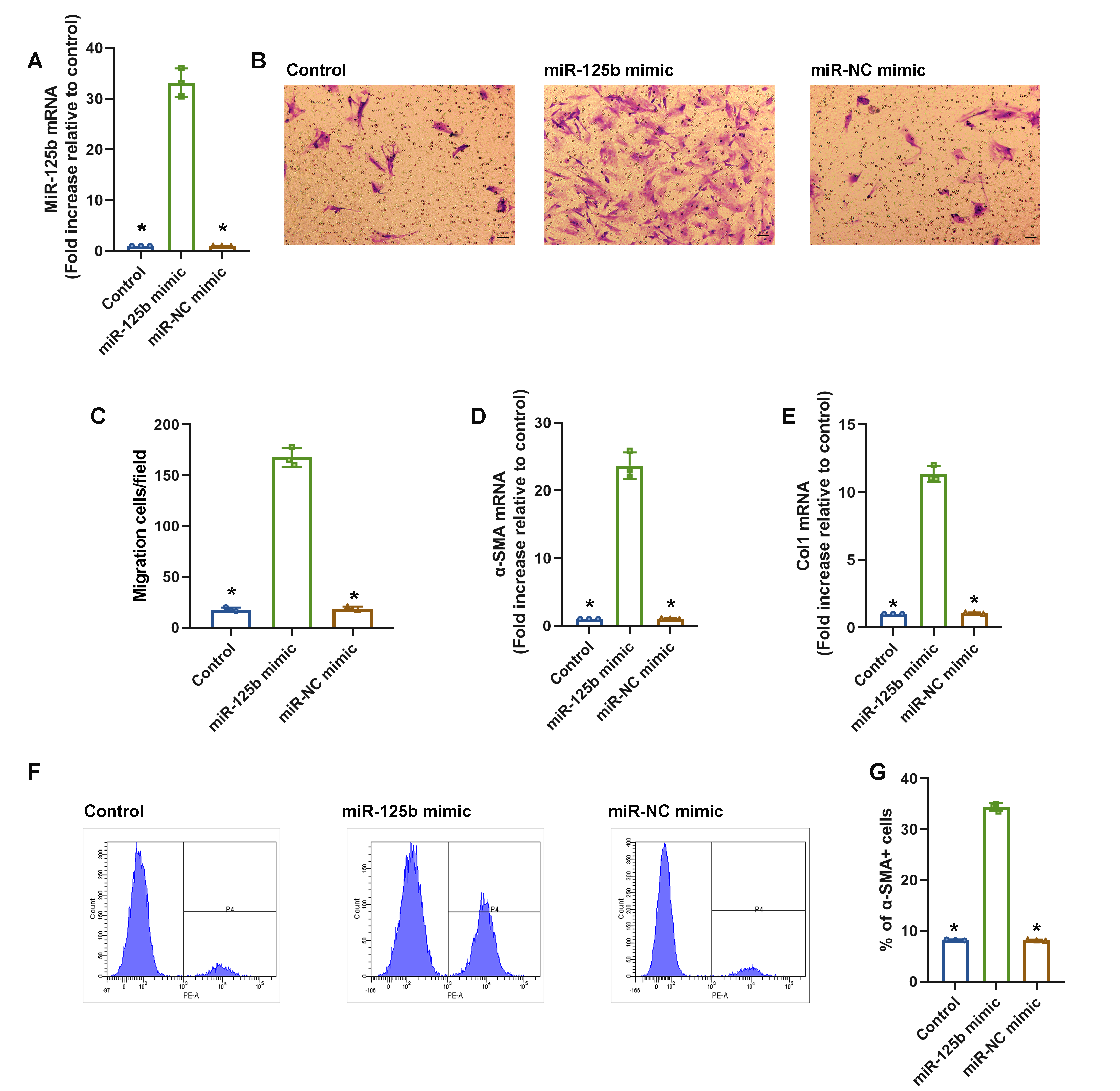

Supplement: Supplementary file 1 — Additional file 1: Fig. S1. MiR-125b promoted fibroblast migration and FMT. A MiR-125b mRNA in fibroblastOld after transfecting miR-125b mimic or miR-NC mimic was examined using qRT-PCR. *P < 0.05 versus miR-125b mimic in repeated measures analysis of variance (n = 3). B Images of migrated fibroblastsOld using Transwell migration assays. C Data are presented as the number of migrated cells. D, E The mRNA levels of the pro-fibrotic genes α-SMA and Col1 were analyzed using qRT-PCR. F FACS plots detailing the gating strategy to define SMA-positive subpopulations. G Quantification of the relative abundance of SMA-positive cells. *P < 0.05 versus miR-125b mimic in repeated measures analysis of variance (n = 3). [file 12951_2022_1348_MOESM1_ESM.tif]

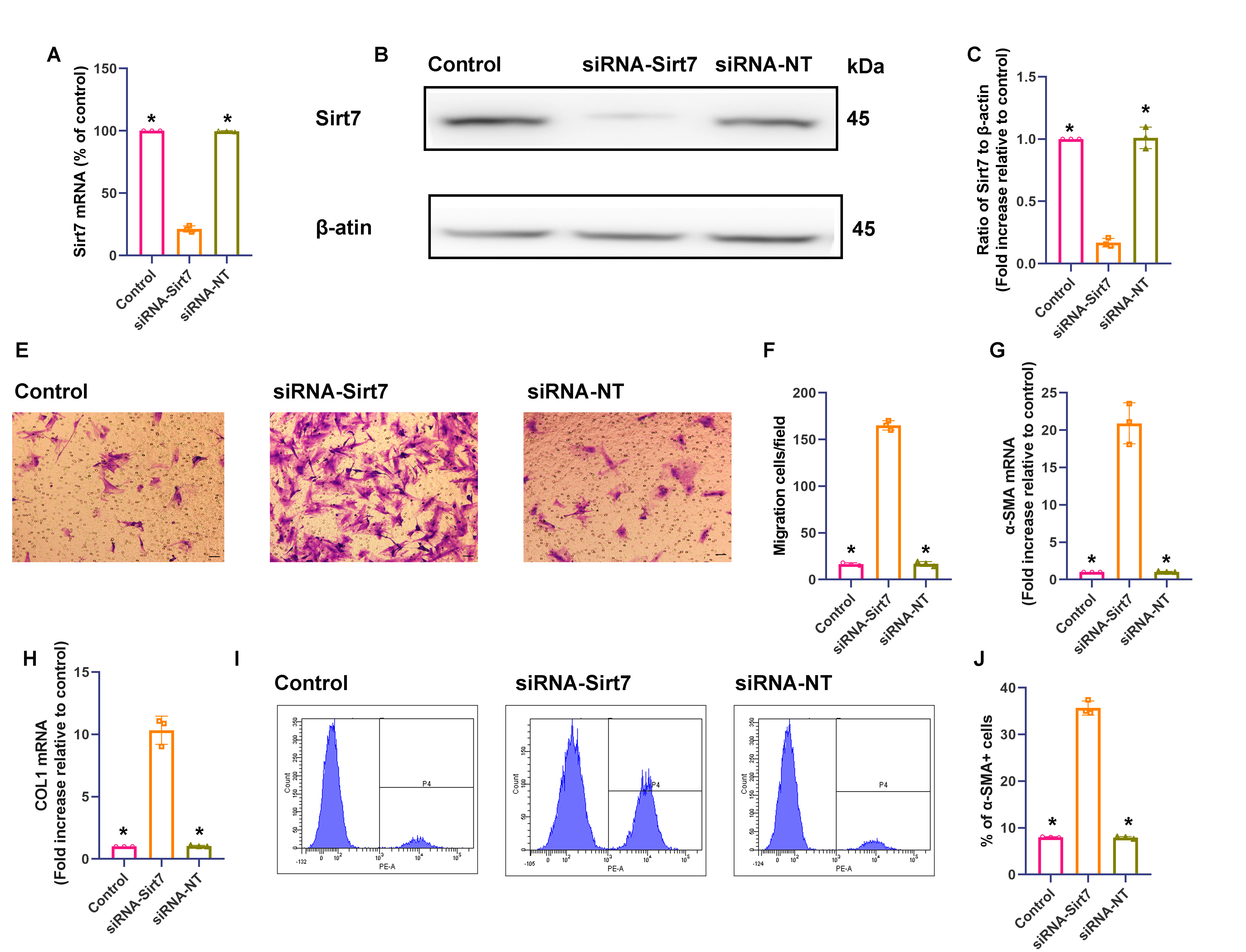

Supplement: Supplementary file 2 — Additional file 2: Fig. S2. Silencing Sirt7 promoted fibroblast migration and FMT. A–C QRT-PCR A and western blot analysis B,C tested the siRNA-mediated transfection efficiency. D Images of migrated fibroblastsOld using Transwell migration assays.E Data are presented as the number of migrated cells. F, G The mRNA levels of the pro-fibrotic genes α-SMA and Col1 were analyzed using qRT-PCR.H FACS plots detailing the gating strategy to define SMA-positive subpopulations. I Quantification of the relative abundance of SMA-positive cells. *P < 0.05 versus siRNA-Sirt7 in repeated measures analysis of variance (n = 3). [file 12951_2022_1348_MOESM2_ESM.tif]
